# ERK and Akt signaling pathways are involved in advanced glycation end product-induced autophagy in rat vascular smooth muscle cells

**DOI:** 10.3892/ijmm.2012.891

**Published:** 2012-01-23

**Authors:** PENGFEI HU, DONGWU LAI, PEILIN LU, JING GAO, HONG HE

**Affiliations:** 1 Department of Cardiology, Sir Run Run Shaw Hospital, Zhejiang University School of Medicine, Hangzhou 310016; 2 Department of Neurology, Sir Run Run Shaw Hospital, Zhejiang University School of Medicine, Hangzhou 310016; 3 Department of Cardiology, The Third People's Hospital of Hangzhou City, Hangzhou 31009, Zhejiang Province, P.R. China

**Keywords:** advanced glycation end products, autophagy, vascular smooth muscle cells, receptor for advanced glycation end products, atherosclerosis

## Abstract

Advanced glycation end products (AGEs) play an important role in the proliferation of vascular smooth muscle cells (VSMCs) and accelerate atherosclerosis in diabetic patients. Autophagy, a life-sustaining process, is stimulated in atherosclerotic plaques by oxidized lipids, inflammation and metabolic stress conditions. In our studies, we utilized MTT assays to show that autophagy is involved in AGE-induced proliferation of VSMCs. Furthermore, treatment with AGEs (100 μg/ml) could induce autophagy in a time- and dose-dependent manner in rat aortic VSMCs. These results were further substantiated by electron microscopy and immunofluorescence imaging. Treatment with AGEs activated ERK, JNK and p38/MAPK, but inhibited Akt. Pretreatment with an ERK inhibitor and an Akt activator inhibited AGE-induced autophagy, demonstrating that AGEs induce autophagy in VSMCs through the ERK and Akt signaling pathways. In addition, RNA interference of RAGE decreased autophagy, indicating that RAGE is pivotal in the process of AGE-induced autophagy. Therefore, AGE-induced autophagy contributes to the process of AGE-induced proliferation of VSMCs, which is related to atherosclerosis in diabetes.

## Introduction

Advanced glycation end products (AGEs) result from the Maillard reaction, which is a non-enzymatic, irreversible process (1). Some studies suggest that AGEs accelerate atherosclerosis in type-2 diabetic patients with coronary heart disease (2,3). In diabetic patients, previous studies have shown that proliferation of vascular smooth muscle cells (VSMCs), a key factor in the development of atherosclerotic lesions, are significantly stimulated by the accumulation of AGEs and their interaction with the receptor for advanced glycation end products (RAGE) (4,5). Activation of RAGE not only accelerates early lesion formation but sustains lesion progression in the diabetic apoE-null mouse model (6,7).

Autophagy is an evolutionarily conserved process involving degradation of long-lived proteins. It is important for balancing sources of energy at critical times (8,9). There are many genes involved in the process of autophagy. Among them, the microtubule-associated protein 1 light chain 3 (LC3) is critical to autophagosome formation. When autophagy is induced, the cytoplasmic form of LC3 (LC3-I) becomes membrane-associated (LC3-II). LC3-II has been used extensively as a marker protein for autophagy (10). In advanced atherosclerotic plaques, autophagy is notably activated by several pathological conditions, such as oxidized lipids, inflammation, oxidative stress and metabolic stress conditions (11–13). However, the relationship between AGEs and autophagy in atherosclerotic plaques is rarely reported. Therefore, we hypothesized that autophagy is a pathological mechanism involved in AGE-accelerated atherosclerosis, especially in AGE-mediated proliferation of VSMCs.

Autophagy involves both the Akt and mitogen activated protein kinase (MAPK) pathways. The ERK pathway, as one of MAPK family members, phosphorylates the Gα-interacting protein to accelerate the rate of GTP hydrolysis to induce autophagy (14). In response to starvation, inflammation and oxidative stress, the PI3K/Akt/mTOR pathway negatively regulates autophagy in VSMCs (15).

The relationship between AGEs and autophagy has not been fully elucidated. To study the underlying mechanisms of AGE-induced autophagy, we examined the activation and the function of autophagy in rat A7R5 VSMCs treated with AGEs.

## Materials and methods

### Materials

Monoclonal rabbit anti-Beclin-1 antibody was obtained from Epitomics (CA, USA). Polyclonal rabbit anti-LC3B antibody was purchased from Novus (CO, USA). Monoclonal rabbit antibodies including anti-Erk, anti-phospho-Erk, anti-JNK, anti-phospho-JNK, anti-p38 and anti-phospho-p38, anti-AKT, anti-phospho-AKT, anti-mTOR and anti-phospho-mTOR were obtained from Cell Signaling Technology (MA, USA). Polyclonal rabbit anti-RAGE antibody was purchased from Millipore (Boston, MA, USA). The MTT, BSA, 3-MA, MAPK inhibitors including PD98059, SP600125 and SB203580, were obtained from Sigma (St. Louis, MO, USA). HRP-marked anti-GAPDH antibody was purchased from Kangchen (Shanghai, China). Rat IGF-1 was obtained from R&D (MN, USA). RAGE RNAi was designed and purchased from Shanghai GenePharma (Shanghai, China).

### Cell culture

Rat A7R5 vascular smooth muscle cells (BioHermes, China) were cultured in low-glucose Dulbecco's modified Eagle's medium (Gibco, USA) supplemented with 10% fetal bovine serum (Sijiqing, China) in a humidified 5% CO_2_/95% air atmosphere at 37°C.

### Preparation of AGEs

AGEs were prepared as previously reported (16). Briefly, BSA was incubated with 0.5 M glucose in phosphate-buffered saline (PBS) in the dark for 16 weeks at 37°C. The unincorporated sugars were removed by dialyzing against PBS (pH 7.4). Control nonglycated BSA was incubated in the absence of glucose under the same conditions. Endotoxin levels were checked using an endotoxin testing kit (Chromogenic TAL Endpoint Assay kit, China). The AGE-BSA solutions were confirmed to be endotoxin free (<2.5 U/ml of endotoxin).

### Western blot analysis

Cells were solubilized in a lysis buffer containing 50 mM Tris (pH 7.4), 150 mM NaCl, 1% NP-40, 5% deoxycholic acid, 0.1% SDS, 1 mM EDTA, 10 mM NaF, 1 mM Na_3_VO_4_, 1 mM dithiothreitol, 1 mM PMSF, 2 μg/ml leupeptin for 30 min. Total protein concentrations were measured by a BCA Protein Assay kit (Applygen Technologies Inc., China). After the samples were heat-denatured, they were analyzed on a 10 or 15% trisglycine gradient gel, transferred to PVDF membranes and blocked with 5% nonfat milk in Tris-buffered solution (TBS) for 1 h at room temperature. The membranes were incubated with primary antibody overnight at 4°C. After being washed three times, membranes were incubated with secondary antibodies for 1.5 h at room temperature. Antibodies were detected by enhanced chemiluminescence (ECL) reagents and imaged using an Image Quant LAS-4000 (Fujifilm, Tokyo, Japan). The band densities were determined using Multi-Gauge Software (Fujifilm).

### Immunofluorescence

Cells were fixed with 4% paraformaldehyde at room temperature for 15 min. After washing with PBS three times, cells were permeabilized with 0.25% Triton X-100 in PBS for 5 min, and then incubated in a blocking buffer containing 10% goat serum, followed by incubation with anti-LC3 antibody (1:200) in PBS containing 10% goat serum overnight at 4°C. After incubating with 0.1% DAPI for 5 min at room temperature and another washing step with PBS, secondary Rhodanmine Red-X labelled antibody (1:100) was applied for 60 min. After washing with PBS, coverslips were transferred onto glass slides. Images were captured on a wide-field fluorescent microscopy (Zeiss).

### Electron microscopy

Ultrastructural analysis was performed to examine autophagy. Rat A7R5 VSMCs were grown in 6-well plates, treated with 100 μg/ml AGEs for 6 h, fixed with a solution containing 3% glutaraldehyde and then sent to Zhejiang University for electron microscopic analysis.

### RNA interference

For function-blocking experiments, we used small interfering RNA molecules (siRNA) targeted to RAGE mRNA. RAGE siRNA was purchased from Shanghai GenePharma. The siRNA was designed against RAGE (sense, 5′-GCCGGAAAUUGUGAAUCCUTT-3′; antisense, 5′-AGGA UUCACAAUUUCCGGCTT-3′). The negative control siRNA was non-targeting (sense, 5′-UUCUCCGAACGUGUCACG UTT-3′; antisense, 5′-ACGUGACACGUUCGGAGAATT-3′). Cells were transfected with si-RAGE using Lipofectamine 2000 (Invitrogen, USA) for 48 h according to the manufacturer's instructions. The final concentration of siRNA was 100 pM. The efficacy of RNA interference was determined by Western blotting.

### Measurement of proliferation of VSMCs

To assess cell proliferation, rat A7R5 VSMCs were plated in a 96-well plate. After 24 h, the medium was changed, and the cells were incubated with fresh medium containing AGEs (100 μg/ml) or 3-MA (2 mM) for another 48 h. Then, 20 μl of MTT solution (final concentration, 5 mg/ml was added to each well for 4 h at 37°C. The medium was then discarded and 100 μl of DMSO was added to each well. The absorbance was measured at 490 nm.

### Statistical analysis

All data were obtained from at least 3 individual experiments. Values are expressed as the mean ± SEM. Statistical analysis between groups was performed by one-way ANOVA. The statistical significance was set at p<0.05.

## Results

### AGE-induced autophagy in rat A7R5 VSMCs

**Figure 1:**
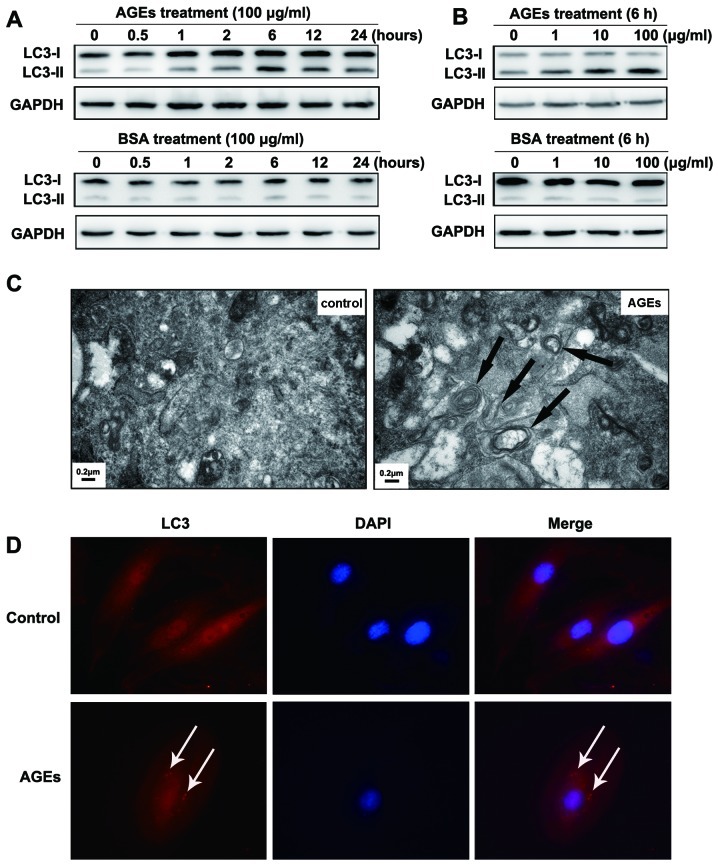
AGE-induced autophagy in A7R5 VSMCs and autophagy is involved in AGE-induced proliferation of VSMCs. (A) Western blot analysis of LC3-I and LC3-II protein levels treated with BSA or AGEs. Cells were treated with 100 μg/ml BSA or AGEs for 0, 0.5, 1, 2, 6, 12 and 24 h. (B) Cells were treated for 6 h with 0, 1, 10, 100 μg/ml BSA or AGEs. The results show a time- and dose-dependent effect of AGEs treatment on the expression of LC3-II and the LC3-II to LC3-I ratio. (C) Representative electron micrographs of VSMCs treated with 100 μg/ml BSA or AGEs for 6 h. Typical autophagic vacuoles containing cellular material or membranous structures (bold arrows) were frequently found in cells treated with AGEs but not BSA. (D) The localization of LC3 as detected by immunofluorescence. In BSA-treated cells, LC3 was distributed homogeneously in the cytoplasm, whereas the cells treated with AGEs showed LC3 dots (red fluorescence) around the nucleus (blue fluorescence).

To determine whether AGEs can affect the level of autophagy in VSMCs, cells were treated with AGEs or BSA (100 μg/ml) for various times (0, 0.5, 1, 2, 6, 12 and 24 h). The expression of LC3-II and the ratio of LC3-II to LC3-I were significantly increased after treatment with AGEs, peaking at 6 h. In contrast, treatment with BSA did not change the expression of LC3-II or the LC3-II to LC3-I ratio (Fig. 1A). Cells were also treated with AGEs or BSA at various concentrations (0, 1, 10 and 100 μg/ml) for 6 h. The expression of LC3-II and the LC3-II to LC3-I ratio were notably increased in a dose-dependent manner in AGE-treated cells (Fig. 1B).

To directly visualize autophagy, we used transmission electron microscopy to examine autophagic vacuoles (autophagosomes). We treated cells with 100 μg/ml BSA or AGEs for 6 h. In BSA-treated cells, autophagic vacuoles were rarely detected. However, we found that autophagic vacuoles containing cellular material or membranous structures were increased in AGE-treated cells (bold arrows in Fig. 1C).

To examine the localization of autophagosomes in rat A7R5 VSMCs, we detected the autophagosome-specific protein LC3 (red fluorescence) by immunofluorescence imaging. Cells were treated with 100 μg/ml BSA or AGEs for 6 h. In BSA-treated cells, LC3 was distributed homogeneously in the cytoplasm. In contrast, LC3 was found in dots around the nucleus in cells treated with AGEs (blue fluorescence) (indicated by white arrows in Fig. 1D).

### Autophagy is involved in AGE-induced proliferation

**Figure 2:**
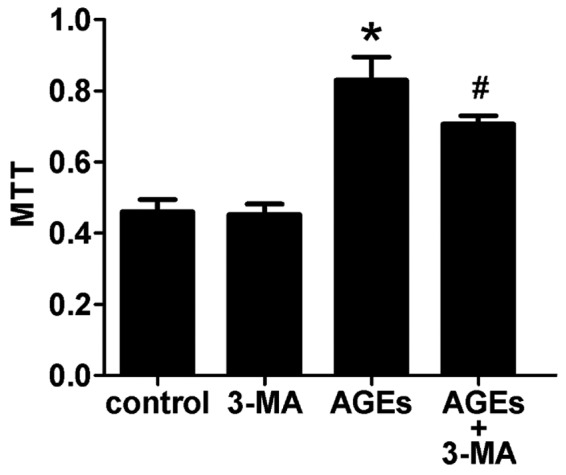
Cell proliferation in cultured A7R5 cells was measured by the MTT assay. Compared with the controls, VSMCs treated with AGEs (100 μg/ml, 48 h) showed increased proliferation. This effect could be attenuated by pretreating cells with the autophagy inhibitor 3-MA. ^*^p<0.05 vs. control; ^#^p<0.05 vs. AGEs; values, means ± SEM.

Compared with the control group, cells treated with 100 μg/ml AGEs for 48 h showed increased proliferation of VSMCs. Furthermore, pretreating cells with 3-MA, an autophagy inhibitor, for 30 min could attenuate this effect (Fig. 2). This indicates that autophagy is involved in AGE-induced proliferation of VSMCs.

### The ERK pathway is involved in AGE-induced autophagy

**Figure 3:**
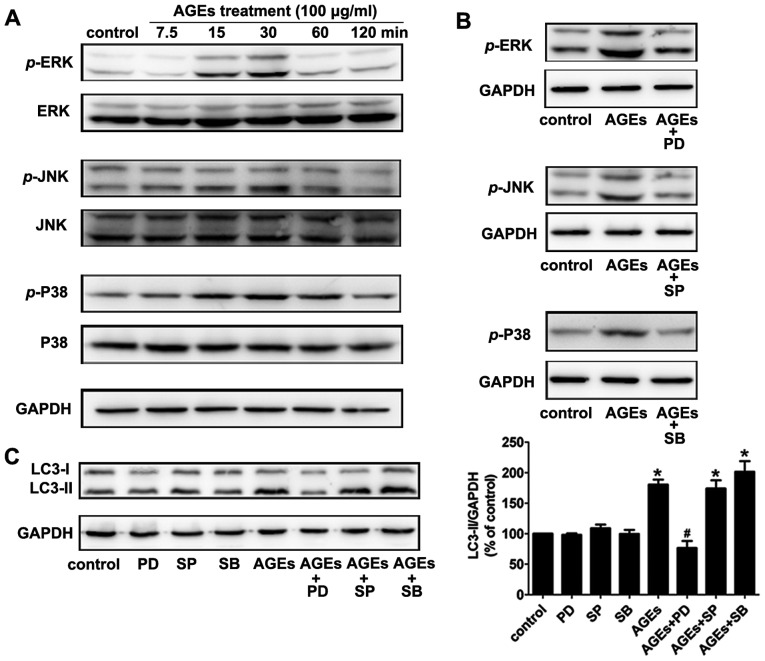
The ERK pathway is involved in AGE-induced autophagy. (A) Western blot analysis of MAPKs in Rat A7R5 VSMCs treated with AGEs. Cells were treated with 100 μg/ml AGEs for the indicated times (7.5, 15, 30, 60 and 120 min). AGEs stimulated phosphorylation of ERK, p38 and JNK in a time-dependent manner. Phosphorylation peaked at 15–30 min and then declined. (B) Western blot of the expression of MAPKs in A7R5 VSMCs pretreated with MAPK inhibitors for 30 min. (C) Western blot analysis of the expression of LC3 in cells treated with AGEs (100 μg/ml, 6 h) in the presence of MAPK inhibitors. The ERK inhibitor PD98059, but not the p38 inhibitor SB203580 and JNK inhibitor SP600125, suppressed AGE-induced expression of LC3-II. These results indicate that ERK signaling is involved in AGEs-induced autophagy. The relative levels of LC3-II to GAPDH are indicated below the corresponding bars. ^*^p<0.05 vs. Control; ^#^p<0.05 vs. AGEs; values, means ± SEM.

MAPK pathways are important for cells to respond to numerous extracellular signals. To investigate the mechanisms involved in AGE-induced autophagy, we examined the phosphorylation level of various MAPK family proteins (ERK, p38, JNK) in VSMCs by western blotting. Cells were treated with 100 μg/ml AGEs for 0, 7.5, 15, 30, 60 and 120 min. As is shown in Fig. 2, AGEs stimulated phosphorylation of MAPKs (ERK, p38, JNK) in rat A7R5 VSMCs in a time-dependent manner. Phosphorylation peaked at 15–30 min and then declined (Fig. 3A). Pretreating cells with the ERK inhibitor PD98059 (20 μM) for 30 min blocked 90% of ERK activation. The p38 MAPK inhibitor SB203580 (10 μM) and JNK MAPK inhibitor SP600125 (20 μM) also blocked the activation of the corresponding MAPKs (Fig. 3B).

In our experiments, the ERK inhibitor PD98059, but not the p38 inhibitor SB203580 or JNK inhibitor SP600125, suppressed the AGE-induced expression of LC3-II (Fig. 3C). The results indicate that the ERK signal transduction pathway is involved in AGE-induced autophagy.

### The Akt/mTOR signaling pathway is involved in AGE-induced autophagy

**Figure 4:**
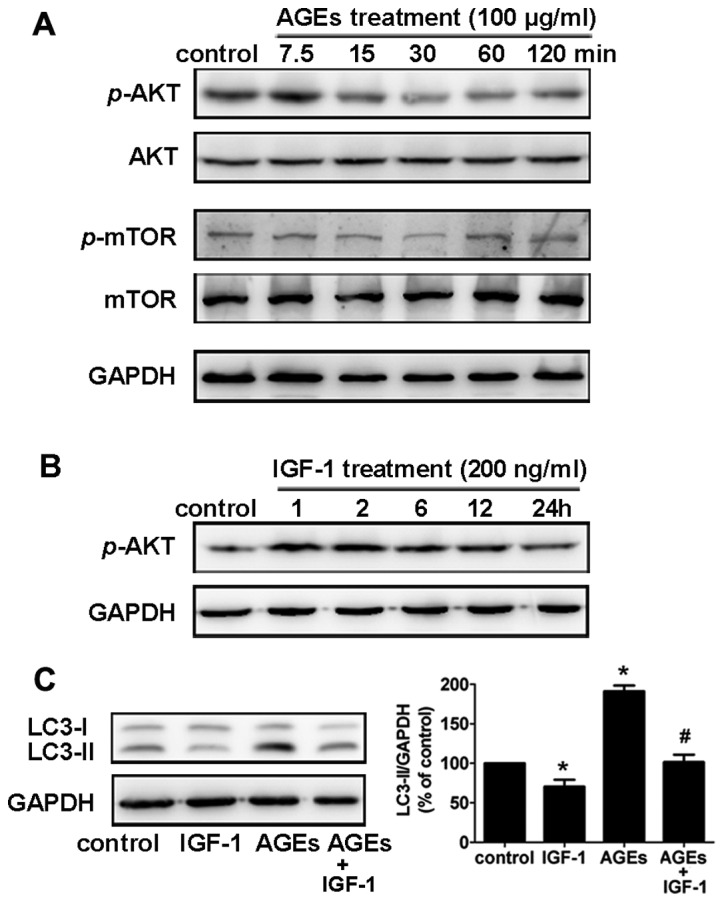
The Akt/mTOR signaling pathway is involved in AGE-induced autophagy. (A) Western blot analysis of the Akt/mTOR pathway in rat A7R5 VSMCs treated with AGEs. In rat A7R5 VSMCs, phosphorylation of Akt and mTOR decreased 30 min to 2 h after treatment with 100 μg/ml AGEs. (B) Insulin-like growth factor 1 (IGF-1) was used to activate the Akt pathway. Cells pretreated with IGF-1 (200 ng/ml) for 1 h showed a notable increase in Akt phosphorylation as detected by western blot analysis. (C) Western blot analysis of LC3 expression in AGE- (100 μg/ml, 6 h) and IGF-1-treated cells. Cells pretreated with IGF-1 (200 ng/ml) suppressed the AGE-induced expression of LC3-II in rat A7R5 VSMCs. The relative levels of LC3-II to GAPDH are indicated below the corresponding bars. ^*^p<0.05 vs. Control; ^#^p<0.05 vs. AGEs; values, means ± SEM.

The Akt/mTOR signaling pathway, which promotes cell growth and survival in response to mitogenic signals, is a major pathway that negatively regulates autophagy. In rat A7R5 VSMCs, phosphorylation of Akt and mTOR decreased 30 min to 2 h after treatment with 100 μg/ml AGEs (Fig. 4A). To examine the role of the Akt/mTOR pathway in AGE-induced autophagy, we used insulin-like growth factor 1 (IGF-1) to activate the Akt pathway (17). We found that rat A7R5 VSMCs pretreated with IGF-1 (200 ng/ml) for 1 h showed a notable increase in Akt phosphorylation (Fig. 4B).

In addition, pretreatment with IGF-1 (200 ng/ml) suppressed the AGE-induced LC3-II expression in Rat A7R5 VSMCs (Fig. 4C). This result suggests that the Akt/mTOR signaling pathway is involved in AGE-induced autophagy in rat A7R5 VSMCs.

### RAGE plays an essential role in AGE-induced autophagy

**Figure 5:**
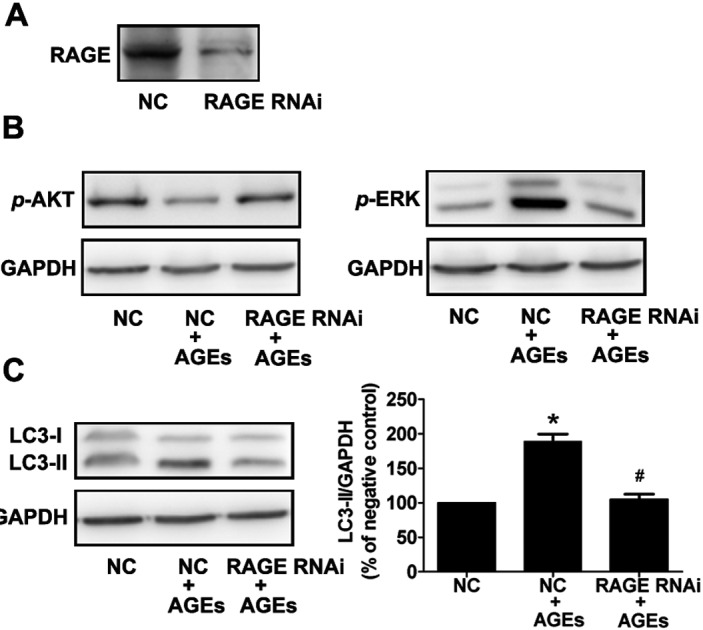
RAGE plays an essential role in AGE-induced autophagy. (A) Protein levels of RAGE, as detected by western blot analysis, are reduced by 90% in cells transfected with siRNA to RAGE compared with control (scrambled) siRNA. (B) Western blot analysis of p-ERK and p-AKT in cells treated with AGEs (100 μg/ml, 6 h) in the presence of RAGE RNAi for 48 h. The activation of ERK was decreased and inhibition of Akt was reversed by RNA interference for RAGE. (C) Western blot analysis of LC3 expression in cells treated with AGEs (100 μg/ml, 6 h) in the presence of RAGE RNAi for 48 h. The expression of LC3-II protein was significantly reduced in RAGE siRNA-transfected cells compared with scrambled control siRNA-transfected cells. The relative levels of LC3-II to GAPDH are indicated by the corresponding bars on the right. ^*^p<0.05 vs. NC (negative control); ^#^p<0.05 vs. NC + AGEs; values, means ± SEM.

To further demonstrate the importance of RAGE in AGE-induced autophagy, cells were treated with siRNA to RAGE, and the levels of activated ERK, Akt, and LC3-II were detected by western blot analysis using phosphospecific antibodies. Compared to the control (scrambled) siRNA-transfected cells, cells transfected with RAGE siRNA exhibited a 90% reduction in RAGE protein expression (Fig. 5A). In AGE-treated cells, activation of ERK and inhibition of AKT was reversed by RNA interference of RAGE (Fig. 5B). This result indicates that AGEs activate ERKs and suppress Akt via RAGE.

Furthermore, we found that the AGE-induced expression of LC3-II was significantly reduced in cells transfected with RAGE siRNA compared with control transfected cells (Fig. 5C). These data illustrate that RAGE plays an essential role in AGE-induced autophagy in A7R5 VSMCs.

## Discussion

Previous studies have demonstrated that AGE-induced proliferation of VSMCs is a key factor in the pathogenesis of acceleration of atherosclerosis in diabetic patients (4,18). Yoon *et al* reported previously that AGEs increased proliferation of VSMCs via ERK and p38 dependent pathways (19). Several recent experimental studies suggest the proliferation of VSMCs in diabetic models is related to several cytokines and growth factors, including platelet derived growth factor (PDGF) (20) and basic fibroblast growth factor (bFGF) (21). In this study, we found that autophagy is also involved in AGE-induced proliferation of VSMCs. The interaction between AGEs and RAGE significantly increased autophagy in VSMCs via the ERK and Akt pathways. This may contribute to proliferation of VSMCs in the pathophysiological process of atherosclerosis in diabetic patients.

Several recent studies have attempted to elucidate the pathological mechanisms underlying disorders that involve AGEs. AGEs have been shown to induce proliferation and migration of VSMCs, increase generation of reactive oxygen species (22), decrease nitric oxide bioavailability (23) and up-regulate the production of various cytokines or growth factors (24,25), such as TNF-α, PDGF and VCAM-1. However, there is no direct evidence for a relationship between AGEs and autophagy. In our study, we found the expression of the autophagosome specific isoform LC3-II protein was enhanced in a time- and dose-dependent manner in cells treated with AGEs. In addition, we observed autophagic vacuoles in AGE-treated cells by transmission electron microscopy. Our results demonstrate that AGEs could induce autophagy, suggesting a novel, pathobiological function for AGEs in diabetes.

Autophagy has been recognized as a cellular defense mechanism that is used to remove protein aggregates and damaged organelles (26). The autophagic vacuole, or autophagosome, contains portions of the cytoplasm and organelles and is surrounded by multiple membrane layers. In the fibrous caps of atherosclerotic plaques, autophagic vacuoles have been detected by electron microscopy in VSMCs (27). However, it is still not clear whether autophagy is beneficial or detrimental in atherosclerotic plaques. In this study, the autophagy inhibitor, 3-MA, could attenuate the effects of AGE-induced proliferation of VSMCs. This result indicates that AGE-induced autophagy is involved in AGE-stimulated proliferation of VSMCs. This may provide a new therapeutic strategy for preventing atherosclerosis in diabetic patients.

RAGE is a major receptor for AGEs. The binding of AGEs to RAGE leads to activation of cell signaling pathways, such as the MAPK, p21_ras_, NF-κB, and JAK/Stat pathways (28,29). We found that AGEs induced phosphorylation of ERK, JNK, and p38 and inhibited phosphorylation of Akt. Furthermore, autophagy in A7R5 VSMCs treated with AGEs was reduced when cells were pretreated with the ERK inhibitor PD98059 but not by SB203580 or SP600125. Activation of the Akt pathway using IGF-1 inhibited AGE-induced autophagy. These results imply that the ERK and Akt pathways have opposing functions in AGE-induced autophagy: the ERK pathway positively regulates autophagy whereas the Akt pathway negatively regulates autophagy. However, since we could not exclude the possibility that other signaling pathways participate in autophagy, further investigation is needed. We also found that RNAi of RAGE in VSMCs inhibited AGE-induced ERK and LC3-II activity and recovered Akt activity. These findings further underscore the importance of the interaction between AGEs and RAGE in AGE-induced autophagy.

In summary, our studies demonstrate that AGEs could induce proliferation of VSMCs through mechanisms involving regulation of autophagy through the ERK and Akt signaling pathways. This suggests that regulating the AGEs-RAGE-autophagy pathway can attenuate proliferation of VSMCs and therefore may reduce the development of atherosclerosis in diabetic patients. Further studies are needed to dissect the relationship between AGEs and autophagy in animal models and to explore possible drug-targeting methods to regulate this pathway.
